# A Novel High‐Throughput Anaerobic Culture Method to Evaluate Food Health Effects via Gut Microbiota Modulation

**DOI:** 10.1002/fsn3.70978

**Published:** 2025-09-18

**Authors:** Xin‐Yue Liu, Ling‐Qin Zhu, Qun‐Ying Jin, Hua‐Zheng Peng, Tang‐Jun Zhu

**Affiliations:** ^1^ College of Forestry and Biotechnology Zhejiang Agriculture and Forestry University Hangzhou China; ^2^ Zhejiang Academy of Forestry Hangzhou China; ^3^ Key Laboratory of National Forestry and Grassland Administration on Forest Food Resources Utilization and Quality Control Beijing China

**Keywords:** bacteria growth detection, intestinal bacteria, microbial fermented product, nutrition evaluation, pecan kernel

## Abstract

As health demands grow, nutritional evaluation now extends beyond nutrient supply, and health‐effect–based methods are gradually gaining traction. However, assessing food impacts on gut microbiota remains complex and costly. This study developed a cost‐effective in vitro method for evaluating food effects on individual gut microbiota, integrating 96‐well microplates, multilayer liquid sealing, and high‐throughput multiomics (16S rRNA sequencing + untargeted metabolomics) to enable large‐scale anaerobic culture and dynamic growth monitoring under aerobic conditions. Using this method, the fermented products of *Pichia fermentans* were shown to significantly boost *Bifidobacterium* abundance (4.6‐fold to 5.6‐fold vs. control) by Day 4, though microbial diversity decreased. Testing raw (RA) versus cooked (CO) pecan kernels, RA significantly promoted health‐associated genera: *Blautia*, which showed progressive enrichment (rising 17‐fold from Day 2 to Day 4 in RA) and reached 193.8 times the control level (5.1%, *p* = 0.0127), with potential anti‐inflammatory implications; *Fusicatenibacter*, surging to 1274.3 times the control (3.3%, *p* = 0.001) and 5.6 times higher than CO (*p* = 0.0332), suggesting potential cognitive benefits; and *Bacteroides*, whose reduction was significantly attenuated in RA compared to control (*p* = 0.0002), potentially supporting bile acid metabolism relevant to ulcerative colitis management. In contrast, CO favored opportunistic pathogens: *Raoultella* was exclusively elevated in CO relative to both control and RA (*p* < 0.05); *Klebsiella* abundance was notably higher in CO than in RA (*p* < 0.05), though increased in both groups versus control. Untargeted metabolomics indicated RA upregulated C5‐branched dibasic acid metabolism and secondary bile acid biosynthesis, accumulating metabolites (e.g., CLA, 7‐KDA) associated with these pathways. This method aids in optimizing food processing and guiding precision nutrition strategies.

## Graphical abstract

We developed a high‐throughput anaerobic culture method (96‐well microplates + multilayer liquid sealing) to evaluate food effects on gut microbiota. Using this method, the fermented products of *Pichia fermentans* were found to boost *Bifidobacterium* abundance (4.6–5.6 × vs. control by Day 4), while raw pecan kernels promoted health‐associated genera (*Blautia*: 193.8 × control; *Fusicatenibacter*: 1274.3 × control, 5.6 × higher than cooked kernels) and upregulated C5‐branched dibasic acid/secondary bile acid metabolism, whereas cooked kernels favored opportunistic pathogens (*Raoultella, Klebsiella*).

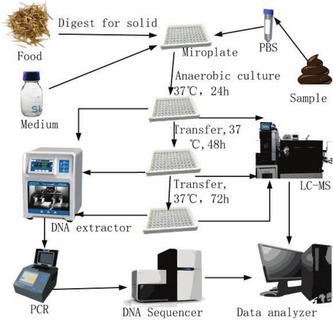

## 1 Introduction

Performing appropriate nutritional evaluations holds significant value for the selection of suitable foods, especially during critical growth stages or during recovery from illness. Traditionally, nutritional assessments of food primarily focus on the type and quantity of nutrient components using nutrition analysis and sensory perception. With the increasing demand for health, such as obesity control, inflammation inhibition, and intellectual development, nutrition nowadays is not limited to the supply of nutrients, but is also an opportunity to prevent the onset of disease and maintain health (Wang, Huang, et al. 2022). In order to achieve the aforementioned objectives, evaluation methods based on the health effects and mechanisms of food are gradually gaining attention, such as cell experiments, animal experiments, and human trials. However, these evaluation methods are often constrained by challenges such as complex experimental procedures, high costs, and lengthy study durations (Wang, Huang, et al. 2022). Additionally, different populations or even individuals usually have varied responses to the same nutrients, further increasing the complexity and cost of assessing the nutritional value.

The intestine serves as both the primary organ for nutrient absorption from dietary sources and the largest immune organ in the human body. Gut microorganisms are critical for the utilization of dietary nutrients and the mediation of food‐derived bioactivities, and the pivotal role of gut microbiota in maintaining human health has been well established (Ross et al. 2024). Therefore, using the response of gut bacteria to food to evaluate its nutritional value has important reference value for judging the health effects of food and its correlation with diseases. For example, recently, to quantify individuals' diets in terms of attaining a healthy gut microbiota, a novel dietary index for gut microbiota (DI‐GM) was developed and compared to existing dietary indices based on its strength in association with indirect biomarkers of gut microbiota diversity using data from the National Health and Nutrition Examination Survey (NHANES) (Kase et al. 2024). However, compared to the diversity of food types and processing methods, studies on their relationship with gut microbiota remain limited due to the many limitations of testing the gut microbiota in vivo.

Due to the difficulty in directly detecting the intestinal microbiota, in vitro culture and testing are the valuable strategies for evaluating the impact of food on the bacteria. However, it is difficult to operate, culture, and detectda the gut microbiota in a conventional aerobic environment since these bacteria are sensitive to oxygen. The commercial anaerobic workstation is a reliable device for the treatment of anaerobic microorganisms (Maier et al. 2018). However, the purchase and maintenance costs of such devices remain high, and their operation is also cumbersome. Therefore, simple anaerobic operation methods or devices are an area of concern, especially for laboratories with limited economic conditions. For example: (i) Hungate's anaerobic technology pioneered pre‐reduced media and nitrogen‐driven oxygen removal in roll tubes, enabling small‐scale anaerobic culture (Hungate 1969); however, its low throughput (typically < 10 samples per experiment) and manual gas‐flushing per sample limit scalability; (ii) Filling station‐based methods automate medium degassing via gas‐flushing systems, improving efficiency (O'Brien and Malvankar 2016; Speers et al. 2009); yet, they rely on specialized equipment (gas tanks, pressure regulators), restricting portability; (iii) Low‐cost anaerobic chambers use oxygen‐absorbing sachets (e.g., iron‐based) to maintain hypoxic environments (Hong et al. 2021; Saha et al. 2016); however, these systems lack real‐time monitoring capabilities, making dynamic growth detection (e.g., hourly absorbance measurements) impractical. Collectively, these methods either suffer from high costs (workstations), low throughput (Hungate), equipment dependency (filling stations), or limited monitoring capacity (anaerobic chambers), hindering large‐scale and longitudinal studies of gut microbiota.

This study proposed a novel in vitro method for detecting the effects of food on individual gut microbiota, which solved the continual growth detection of anaerobic microorganisms in large quantities under conventional aerobic conditions by using a 96‐well microplate, multilayer liquid seal, chemical adsorption, and reasonable operation methods. Using this method, the effects of different food forms on the gut microbiota of the same individual were compared, including liquid yeast fermented products and digested pecan fruits consumed in different ways. By analyzing the abundance changes and metabolic composition of these microbiota over time, the potential health benefits of food were revealed, as well as the impact of different processing methods on food function.

## 2 Materials and Methods

### 2.1 Samples, Strains, and Medium

*Bifidobacterium longum* , *Clostridium butyricum* , and *Enterobacter aerogenes* were isolated from the feces of healthy volunteers (age < 28 years) and identified by 16S rRNA sequencing. Fecal samples were collected from volunteers who had not used antibiotics in the past 12 months and were confirmed healthy via written informed consent. The labels of fecal samples were anonymized using randomly generated alphanumeric codes, with no personal identifiers recorded. Yeast extract–Casitone–Fatty Acid (YCFA) was used as the basic medium, prepared according to standard protocols (Browne et al. 2016).

### 2.2 Food Preparation

The stock solutions of fructose (Sigma, USA) and procyanidin (Sigma, USA) were 5 mM (about 40 times the working concentration) (Santos et al. 2021). The microbial fermented product of *Pichia fermentans* 15B1 (our patented strain, CN202110032614.8, preserved at the China Microbial Culture Collection Center No. 19317) and *P. fermentans* 323 (purchased from the China Industrial Microbial Culture Collection Management Center, *P. fermentans* P3238) was prepared via standard fermentation protocols.

Pecan kernels (cultivar “Mohawk”) were processed into raw (RA) and roasted (CO) forms. For roasting, raw kernels were roasted in a preheated electric blast drying oven at 151°C–171°C for 20–30 min until the seed coat turned scorched brown and emitted a fragrant aroma, achieving a crispy texture. Both raw and roasted pecan kernels were subjected to simulated digestion processes (oral, gastric, and intestinal stages). Oral stage: 5.

g of preliminarily chopped and ground raw or roasted pecan kernels were mixed with 100 mL of sterile water, homogenized in a mortar at a normal speed for 5 min (particle diameter < 1 mm), and incubated with 5 mL α‐amylase solution containing 12 mg α‐amylase (380 U/mg, Nanjing Dole Biology) in 1 mM CaCl_2_ (pH 7.0) at 37°C for 30 min. Gastric stage: the mixture's pH was adjusted to 2.8 with 6 mol/L HCl, and 80 mg of pepsin (> 3000 U/mg, Shanghai Macklin Biochemistry) was added, and the mixture was then incubated at 37°C with shaking (110 rpm) for 2 h. Intestinal stage: the pH was adjusted to 5.5 with 6 M NaOH solution; 10 mL of pancreatin–bile extract solution containing 0.1 g of pancreatin (> 227 U/mg, Shanghai Macklin Biochemistry) and 0.25 g of bile extract (SANGON, Shanghai) in 0.5 M NaHCO_3_ was added, the pH was adjusted to 6.5, and the mixture was incubated at 37°C with shaking at 110 rpm for 3 h.

### 2.3 Strain Culture and Detection

Preparation of oxygen‐removing bag: A 20 cm × 30 cm double‐sealed polyethylene plastic bag was filled with an oxygen absorbent mixture (iron powder: vermiculite: activated carbon: salt = 14:3:2:1, w/w). The bags were prepared in bulk, sealed, and stored at room temperature for up to 6 months. Preparation of gut microbiota: The mixed intestinal bacteria utilized in this study were derived from a single donor's fecal sample. About 1 g of fecal sample was added to 10 mL phosphate buffered saline (PBS) solution (pH 7.0) containing 0.1% (w/v) cysteine (deoxidizer) and vortexed for 1 min to homogenize. Preparation of test microplate: A sterile 96‐well microplate was placed into an oxygen‐removing bag for 24 h to predeoxygenate. A quantity of 150 μL of YCFA medium was added to each well, followed by sequential layering of 20 μL of liquid paraffin, 20 μL of vitamin E, and 20 μL of silicone oil using a multi‐channel pipette. The plate was incubated in the oxygen‐removing bag at 4°C for 24 h before use. When food samples were added, 20 μL of fresh 1% (w/v) cysteine solution was supplemented to each well. The plate was sealed with a lid, returned to the anaerobic bag, and stored at 4°C overnight. Totally, 16 replicates were set for each treatment.

Inoculation of bacteria: Frozen bacterial stocks (−80°C, 1.5 mL cryotube) were thawed at room temperature for 5 min. One hundred microliters of thawed culture was transferred into a 510 mL Penicillin bottle containing 4 mL of YCFA medium and 500 μL of 1% (w/v) cysteine solution. The bottle was sealed with a rubber plug and incubated at 37°C for 16–18 h. Before use, the bottle was vortexed for 10 s, and OD600 was measured using an Ultrospec 10 Cell Density Meter (GE Healthcare). When OD600 ≥ 1.0, 15 μL of culture was inoculated into prepared microplates under an ice bath (4°C). The microplate was sealed with a PCR optical adhesive film to reduce the airflow above the well and incubated in the anaerobic bag at 37°C for 30 min to equilibrate.

Detection and collection of samples: microplates for individual microbial cultivation can be incubated at 37°C in a microplate reader (Varioskan, USA), facilitating automatic detection of absorbance every 1‐2 h. Every 24 h, the microplate containing mixed intestinal microbiota samples in each well was removed from the anaerobic bag, and 15 μL of bacterial suspension was transferred from each well to a new microplate with fresh medium on ice. The remaining bacteria in the original microplates were frozen at −80 degrees for DNA extraction and metabolome analysis. The transfer was repeated to obtain samples at 24 h (Day 2), 48 h (Day 3), and 72 h (Day 4).

### 2.4 DNA Isolation and Sequencing

DNA isolation: For each treatment group, 3–4 wells of bacterial culture (150 μL/well) were collected and transferred to a 1.5 mL microcentrifuge tube. The tube was centrifuged at 12,000 g for 1 min at 4°C, the supernatant was discarded, and the pellet was washed with 150 μL of sterile water 2‐3 times. A quantity of 600 μL of cetyltrimethylammonium bromide (CTAB) extraction buffer (2% CTAB, 100 mM Tris–HCl pH 8.0, 20 mM EDTA, 1.4 M NaCl) and 0.3 g of 0.1 mm zirconia beads were added to the pellet. The mixture was ground in MM400 mixer (Retsch, Germany) at 25 Hz for 5 min, incubated at 65°C for 15 min, and centrifuged at 12,000 g for 1 min. Four hundred microliters of supernatant was transferred to a silica adsorption column, 200 μL of ethanol was added, and the column was centrifuged at 12,000 g for 1 min. The column was washed with 500 μL of 70% ethanol and centrifuged at 12,000 g for 1 min. DNA was eluted with 50 μL of TE buffer (pH 8.0) and stored at −80°C. The V4‐V5 region of 16S rRNA was amplified using primers 515F (GTGCCAGCMGCCGCGGTAA) and 907R (CCGTCAATTCMTTTRAGTTT) with sample‐specific barcodes. PCR reactions (30 μL) were prepared containing 15 μL of Phusion High‐Fidelity PCR Master Mix (NEB), 0.2 μM of each primer, and 10 ng of template DNA. Thermal cycling was performed as follows: 98°C for 1 min (initial denaturation); 30 cycles of 98°C for 10 s (denaturation), 50°C for 30 s (annealing), 72°C for 60 s (extension); final extension at 72°C for 5 min. Sequencing libraries were generated using NEB NextUltraDNA Library Prep Kit for Illumina (NEB, USA) following manufacturer's recommendations, and index codes were added. The library quality was assessed on the Fluorometer (Thermo Scientific) and Agilent Bioanalyzer 2100 system. At last, the library was sequenced on an Illumina MiSeq platform, and 250 bp/300 bp paired‐end reads were generated.

### 2.5 Analysis of Untargeted Metabolomics

Untargeted metabolomics were performed according to the reference (Alseekh et al. 2021). Liquid chromatography–tandem mass spectrometry (LC–MS/MS) analyses were performed using a UHPLC system (Vanquish, Thermo Fisher Scientific) with a Waters ACQUITY UPLC BEH Amide (2.1 mm × 50 mm, 1.7 μm) coupled to an Orbitrap Exploris 120 mass spectrometer (Orbitrap MS, Thermo). The Orbitrap Exploris 120 mass spectrometer was used for its ability to acquire MS/MS spectra in information‐dependent acquisition (IDA) mode under the control of the acquisition software (Xcalibur, Thermo). The ESI source conditions were set as follows: sheath gas flow rate 50 Arb, Aux gas flow rate 15 Arb, capillary temperature 320°C, full MS resolution 60,000, MS/MS resolution 15,000, collision energySNCE 20/30/40 (stepped), spray voltage 3.8 kV (positive), or −3.4 kV (negative).

### 2.6 Data Analysis

Paired‐end reads from the original DNA fragments were merged using FLASH and assigned to each sample according to the unique barcodes. Operational Taxonomic Unit (OTU) clustering and species annotation were performed by the UPARSE software package using the UPARSE‐OTU and UPARSE‐OTUref algorithms. Alpha and beta diversity analyses were conducted using in‐house Perl scripts. Sequences with ≥ 97% similarity were assigned to the same OTUs. Data were further analyzed, and graphs were drawn by Excel and Visio of Office 2010 (Microsoft, USA). Screening criteria for bacteria with the most significant changes: Bacteria were ranked by the ratio of the treatment group to the control group from Day 2 to Day 4. For genera where the maximum mean abundance across groups was > 1% and < 10%, those with a ratio > 10 were selected; for genera where the maximum mean abundance across groups was > 10%, those with a ratio > 2 were selected. Prior to significance testing of microbial community differences, an arcsine normalization transformation was performed to achieve a normal distribution, followed by a two‐tailed t‐test. The raw data of untargeted metabolomics were converted to the mzXML format using ProteoWizard and processed with an in‐house program, which was developed using R and based on XCMS, for peak detection, extraction, alignment, and integration. The R package and the MingkebioDB (V3.0) were first applied in metabolite identification. Subsequent metabolomic analyses, including PCA, PLS‐DA, metabolic pathway classification, and DA‐Score calculation, were performed using Python (version 3.12). Significance *p* values were derived from two‐tailed t‐tests; prior to testing, metabolomic data were normalized and log‐transformed to satisfy the assumptions of a normal distribution. The DA score was calculated as follows: DA score = −log_10_ (*p* value of pathway) × median (log_2_FC of metabolites within the pathway). Differential metabolites were identified based on the criteria of |log_2_FC| > 1, *p* < 0.05, and VIP (variable importance in projection) > 1.

## 3 Results and Discussion

### 3.1 Validation of the Novel Method for Individual Gut Bacteria Culture

**FIGURE 1:**
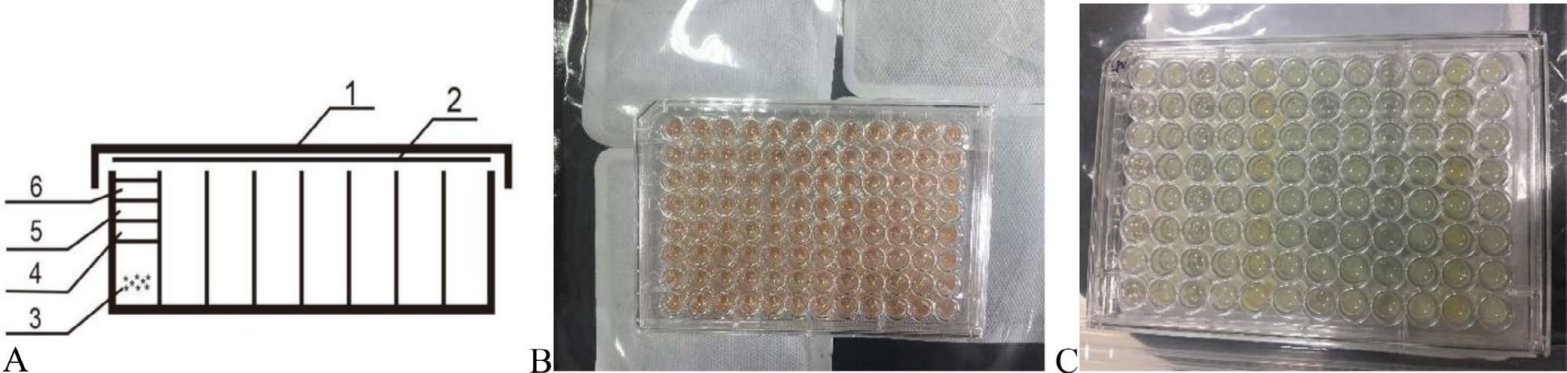
(A) Design of microplate device for anaerobic culture: 1. microplate cover, 2. optical film, 3. liquid medium, 4. silicone oil, 5. Ve, 6. liquid paraffin; (B) color of medium before deoxygenation; (C) color after deoxygenation and growth of bacteria.

Since most anaerobic bacteria cannot survive at daily oxygen concentrations and even some facultative anaerobic bacteria have different responses to foods under aerobic and anaerobic conditions (Eini et al. 2013), anaerobic conditions are the key to the cultivation of intestinal bacteria and food testing. The design of the culture device in this study was shown in Figure 1A. There are multiple layers of oily liquid on the liquid medium. Since the density order is liquid paraffin < Ve < silicone oil < water, they are naturally layered from top to bottom when they are added. The silicone oil here can play an important role in ensuring the detection stability of absorbance when detecting the growth of gas‐producing microorganisms. The medium was red before the addition of deoxidizers and test reagents (Figure 1B). After the addition of deoxidizers and reagents, the red faded after the pretreatment of deoxidization in a sealed bag containing deoxidizers (Figure 1C).

**FIGURE 2:**
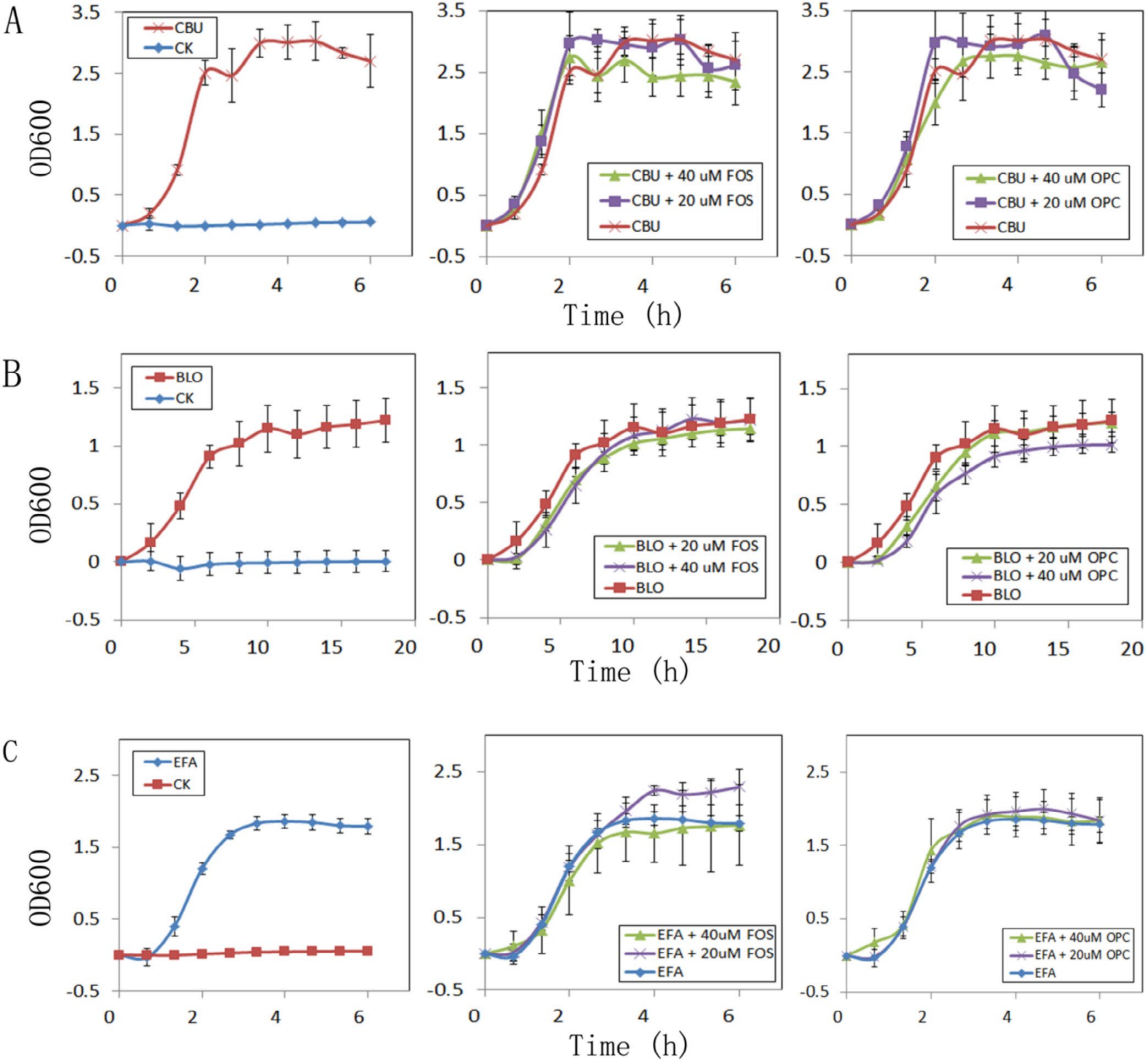
(A) Effects of fructose (FOS) and procyanidin (OPC) on the growth of *C. butyricum* (CBU), CK as a sterile control; (B) Effects of FOS) and OPC on the growth of *B. longum* (BLO), CK as a sterile control; (C) Effects of FOS and OPC on the growth of *E. faecalis* (EFA), CK as a sterile control.

In order to verify the feasibility of intestinal bacteria culture, two strict anaerobic bacteria, *C. butyricum* and *B. longum* , and a facultative anaerobic bacterium, *E. faecalis* , were selected for continuous culture and dynamic absorbance tests. The results showed that the three bacteria had good growth in the microplate, and there was no significant change in absorbance in the sterile control, indicating that this culture method can reflect the growth of the inoculated bacteria reliably (Figure 2). The culture of *C. butyricum* showed growth curves with four stages (Figure 2A): lag phase, logarithmic phase, stationary phase, and death phase. *C. butyricum* completed its rapid proliferation process about 1 h after the lag phase and entered the stable growth stage about 3 h after the detection. The growth pattern of *B. longum* was different (Figure 2B). Its rapid growth stage after the lag phase was about 5 h. After 10 h of detection, it gradually entered the stable stage, but was still slowly growing after 18 h. The growth process of *Enterococcus faecalis* was similar to that of *C. butyricum* (Figure 2C). Its rapid growth time after the lag phase was about 2 h, and the stable phase was about 3 h after detection.

The effects of FOS and OPC on the growth of three strains were also detected. The results showed that the presence of FOS could promote the rapid growth of *C. butyricum* in the logarithmic phase, but the high concentration of FOS could inhibit the growth of *C. butyricum* in the stationary phase. Similarly (Figure 2A), OPC had little effect on the growth of *C. butyricum* . For *B. longum* (Figure 2B), in the rapid growth stage, both high and low concentrations of FOS or OPC have a certain inhibitory effect on its growth, but in the stationary phase, only high concentrations of OPC have a certain inhibitory effect on its growth. For *E. faecalis* (Figure 2C), in the rapid growth stage, both high and low concentrations of FOS or OPC had little effect on its growth, but in the stationary phase, low concentrations of FOS had a certain growth‐promoting effect.

**FIGURE 3:**
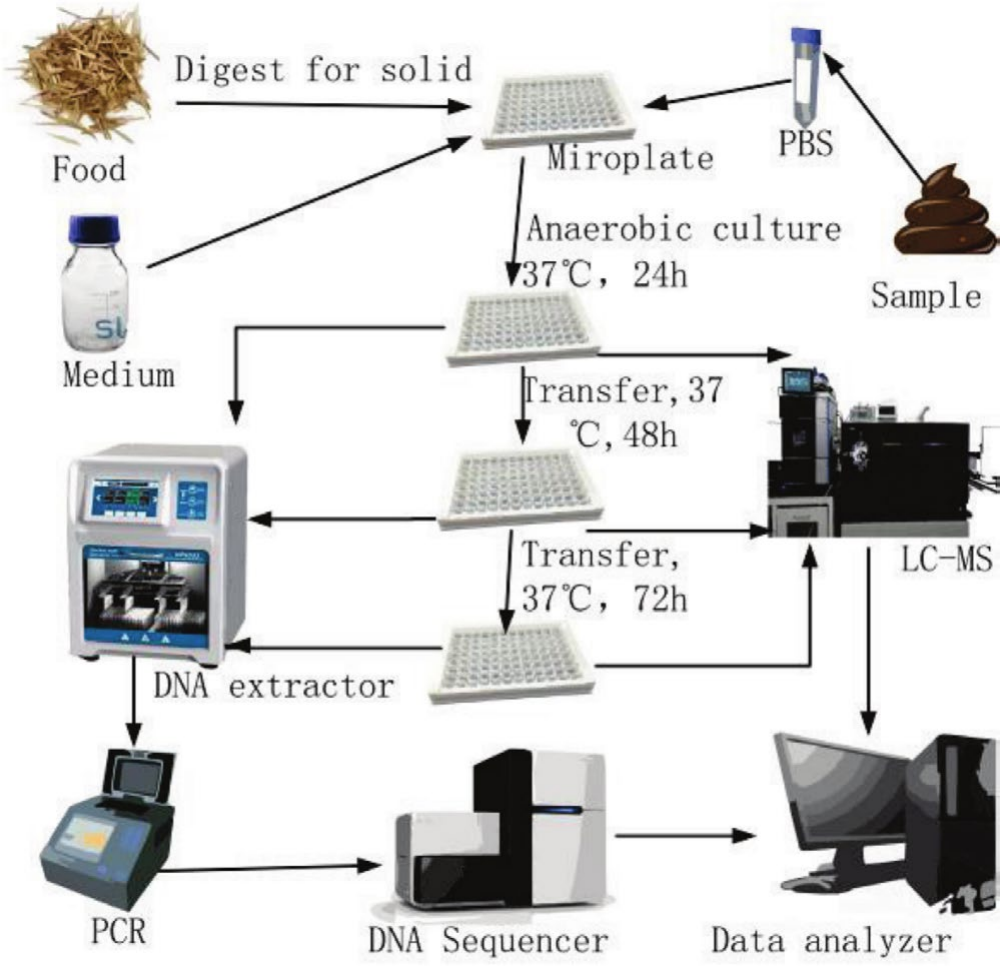
Flow chart of testing foods on gut microbiota.

On the basis of the above‐mentioned individual bacteria test, the method on the mixed intestinal bacteria was designed (Figure 3). This method innovatively integrates multi‐layer liquid sealing (liquid paraffin/vitamin E/silicone oil) and self‐made oxygen‐removal bags to enable large‐scale anaerobic culture in standard microplate readers, overcoming dependence on anaerobic workstations while ensuring reproducibility and biosafety.

### 3.2 Proliferation of *Bifidobacterium* by two *P. fermentans* Strains

Microbial fermented products are often utilized as health beverages. In this experiment, to test whether two yeast strains, *P. fermentans* 15B1 and *P. fermentans* 323, had the ability to regulate intestinal probiotics, their fermentation supernatants were directly added to microplates containing intestinal bacteria to compare the differences in microbiota changes.

After DNA extraction and sequencing of the processed samples, the abundance of microbiota was analyzed at the genus level from three perspectives: dominant bacteria, diversity, and trends of individual bacteria. Examining the differences and changes in the dominant microbiota (> 5%) from Day 2 to Day 4 in each treatment (Table 1), the dominant bacteria in the control group (CK) decreased from four to one, with the most predominant *Streptococcus* increasing from an initial 49% to the last 84%. Notably, the important probiotic *Bifidobacterium*, which was one of the dominant bacteria on Day 2 with a content of 7%, ceased to be dominant on Day 4, indicating a significant imbalance in the microbiota structure (Table 1). In contrast, the dominant microbiota in the two groups treated with 15B1 and 323 remained relatively stable. Although *Streptococcus* remained the most dominant species in both groups, its proportion decreased relatively. For example, in the 15B1 group, the number of dominant bacteria decreased from six on Day 2 to four on Day 4, with the highest *Streptococcus* increasing from 42% to 65%. Notably, *Bifidobacterium* increased from 5% to 16%. Similarly, in the 323 group, *Bifidobacterium*, which was not even a dominant genus on Day 2, eventually increased to 19%. This demonstrated a clear promotional effect of these fermented products on the probiotic *Bifidobacterium*.

**TABLE 1:** Effects of addition of microbial fermented products on composition and structure of dominant bacteria in intestinal microbiota (≥ 5%).

Time	CK		15B1		323	
	Dominant genus	Abundance (Mean ± SD)	Dominant genus	Abundance (Mean ± SD)	Dominant genus	Abundance (Mean ± SD)
Day 2	Str	0.49 ± 0.01	Str	0.42 ± 0.02	Str	0.38 ± 0.02
	Esc	0.17 ± 0.03	Ery	0.16 ± 0.01	Ery	0.16 ± 0.02
	Ery	0.13 ± 0.01	Esc	0.13 ± 0.01	Esc	0.15 ± 0.01
	Bif	0.07 ± 0.01	Col	0.06 ± 0.01	Col	0.07 ± 0.04
			Ere	0.05 ± 0.00	Ere	0.05 ± 0.00
			Bif	0.05 ± 0.00		
Day 3	Str	0.77 ± 0.04	Str	0.55 ± 0.03	Str	0.54 ± 0.02
	Bif	0.09 ± 0.02	Esc	0.16 ± 0.01	Esc	0.17 ± 0.02
	Esc	0.07 ± 0.00	Col	0.12 ± 0.02	Col	0.14 ± 0.02
			Bif	0.11 ± 0.01	Bif	0.10 ± 0.01
Day 4	Str	0.84 ± 0.18	Str	0.65 ± 0.06	Str	0.62 ± 0.03
			Bif	0.16 ± 0.02	Bif	0.19 ± 0.00
			Col	0.12 ± 0.04	Col	0.09 ± 0.03
			Esc	0.05 ± 0.01	Esc	0.07 ± 0.01

Abbreviations: Bif, *Bifidobacterium*; Col, *Collinsella*; Ere, *[Eubacterium] rectale* group, Ery, *Erysipelotrichaceae* UCG‐003; Esc, *Escherichia‐Shigella*; Kle, *Klebsiella*.

**FIGURE 4:**
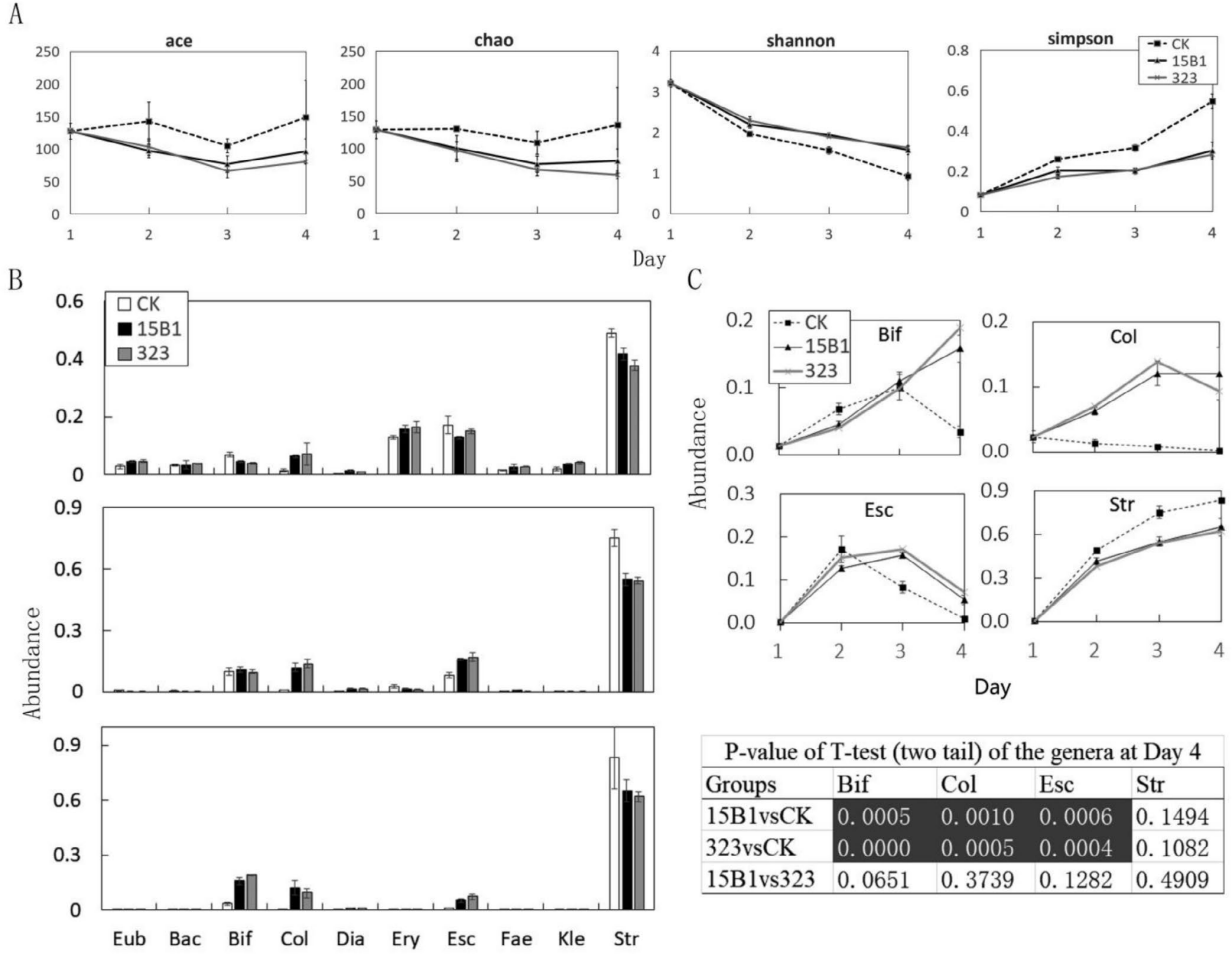
Influence of strains 15B1 and 323 on gut microbiota. (A) The bacterial diversity among groups. (B) The ten genera with the largest variance during the Day 2–Day 4. (C) The abundance variation of four most obvious genera in each treatment. Eub: *Eubacterium*; Bac: *Bacteroides*; Bif: *Bifidobacterium*; Col: *Collinsella*; Dia: *Dialister*; Ery: *Erysipelotrichaceae*; Esc: *Escherichia*; Fae: *Faecalibacterium*; Kle: *Klebsiella*; Str: *Streptococcus*. White text in black background indicated significant differences in the genus between the two groups.

In terms of microbiota diversity, the number of bacteria in the control sample remained stable over 4 days based on Abundance‐based Coverage Estimator (ACE) and Chao1 values, but the Shannon index and Simpson index revealed that its diversity decreased gradually. Compared to the control, the fermentation broths of strains 15B1 and 323 gradually reduced the microbial diversity as well. However, the decline in their microbial diversity was slower, consistent with the analysis of dominant bacteria (Figure 4A). This suggested that the reduction in microbial diversity induced by the fermentation broths may be associated with the baseline medium used. When this factor was taken into account, the addition of fermentation broths actually improved microbial diversity to a certain extent.

Further analysis of the top 10 bacterial genera with the greatest changes showed that the overall change of intestinal microbiota induced by 15B1 and 323 was similar, but there were three significant differences compared with the control (Figure 4B,C). *Bifidobacterium* in the control group increased gradually from Day 1 to Day 3 and decreased sharply on Day 4. In the presence of *P. fermentans* fermented products, the abundance of *Bifidobacterium* increased continuously instead of decreasing on Day 4. The abundance of the 15B1 group was 15.8% on Day 4, which was 4.6 times that of the control (*p* = 0.0005 < 0.01). Similarly, the 323 group was 18.9% on Day 4, which was 5.6 times that of the control (*p* < 0.01). For *Collinsella*, the control group decreased gradually from Day 1 to Day 4, while the 15B1 and 323 groups increased rapidly from Day 1 to Day 3. The final abundances of the 15B1 and 323 groups reached 12.0% and 9.3%, respectively, on Day 4, which were 54.9 times (*p* = 0.001 < 0.01) and 42.5 times (*p* = 0.0005 < 0.01) that of the control, respectively. For the genus *Escherichia*, all the groups reached a maximum abundance on Day 2 or Day 3 but declined rapidly on Day 4. Finally, the abundances of the 15B1 and 323 groups were still higher than that of the control on Day 4, reaching 5.3% (*p* = 0.0006 < 0.01) and 7.1% (*p* = 0.004 < 0.01), respectively.

When comparing the effects of foods in this method, error control within groups was crucial for finding significant increases or decreases of some bacteria after treatments. It was found that most of the errors of bacteria with abundance above 1% were controlled within 10% within the 15B1 and 323 groups, which was equivalent to the error of OD600 from single bacterial growth curves among the wells. The intra‐group error mainly came from the difference in bacterial growth, the difference in DNA extraction, and the difference in sequencing. Although the presence of these errors may reduce the discriminability of different treatments, the different effects brought by treatments will be amplified due to several days of transfer cultivation. The increase of *Bifidobacterium* in this experiment by *Pichia pastoris* 15B1 treatment was not very obvious in the first 2 days, but there was a significant difference on Day 4 between the treatment and control.

The study revealed that both 15B1 and 323 could stimulate the intestinal microbiota of individuals to produce a significant amount of *Bifidobacterium* beneficial to health. This was consistent with some results from animal experiments; for example, yeast fermented products including Saccharomyces and Pichia yeasts can promote the growth of *Bifidobacterium* in dogs (Bastos et al. 2023; Wang, Li, et al. 2022). However, our results also showed that the treatment of yeast fermentation broth for 3 days had a trend of promoting excessive growth of *Streptococcus* bacteria and reducing the diversity of intestinal microbiota. These results provided valuable insights into the relationship between the consumption of fermented products and health.

### 3.3 Effects of Raw versus Cooked Pecan Kernels on Gut Probiotics and Pathogens

Pecan kernels possess significant nutritional value and are associated with potential benefits for inflammatory bowel disease (IBD) and cognitive development (Atanasov et al. 2018). This study compared raw (RA) and cooked (CO) pecan consumption by analyzing their digestates' effects on gut microbiota. Preliminary digestion preceded microplate testing, revealing that both pecan treatments significantly modulated gut microbial composition. From Day 2 to Day 4 (Table 2), the control group (CK) exhibited a reduction in dominant bacterial genera from five to three, with *Streptococcus* dominance increasing sharply from 40% to 65%. Conversely, RA and CO groups maintained stable or slightly increased numbers of dominant genera (Table 2). The RA group was characterized by *Escherichia* (decreasing from 36% to 33%) and two genera absent in CK: stable *Collinsella* and *Blautia*, which emerged uniquely in RA by Day 4. The CO group featured persistent *Collinsella* dominance and three distinctive genera (*Klebsiella*, *Enterobacter*, and *Raoultella*), the latter induced on Day 4.

**TABLE 2:** Effects of addition of pecan kernel on composition and structure of dominant bacteria in intestinal microbiota (≥ 5%).

Time	CK		RA		CO	
	Dominant genus	Abundance (Mean ± SD)	Dominant genus	Abundance (Mean ± SD)	Dominant genus	Abundance (Mean ± SD)
Day 2	Str	0.40 ± 0.05	Esc	0.36 ± 0.06	Col	0.24 ± 0.02
	Ery	0.25 ± 0.05	Col	0.20 ± 0.05	Esc	0.23 ± 0.12
	Bif	0.09 ± 0.01	Ery	0.17 ± 0.02	Bac	0.15 ± 0.04
	Bac	0.06 ± 0.01	Bac	0.12 ± 0.02	Ery	0.11 ± 0.02
	Lac	0.05 ± 0.01			Kle	0.06 ± 0.04
					Ent	0.05 ± 0.06
Day 3	Str	0.61 ± 0.01	Esc	0.36 ± 0.03	Col	0.23 ± 0.05
	Lim	0.11 ± 0.01	Col	0.23 ± 0.05	Esc	0.22 ± 0.13
	Bif	0.09 ± 0.01	Str	0.11 ± 0.04	Str	0.13 ± 0.04
	Col	0.08 ± 0.00	Bac	0.07 ± 0.01	Bac	0.10 ± 0.05
			Ery	0.06 ± 0.02	Kle	0.08 ± 0.08
					Ent	0.05 ± 0.08
Day 4	Str	0.65 ± 0.01	Esc	0.33 ± 0.02	Col	0.19 ± 0.01
	Lim	0.15 ± 0.02	Col	0.16 ± 0.03	Str	0.16 ± 0.05
	Bif	0.06 ± 0.01	Str	0.15 ± 0.05	Esc	0.13 ± 0.12
			Lim	0.10 ± 0.08	Bac	0.10 ± 0.08
			Bla	0.05 ± 0.03	Rao	0.09 ± 0.04
					Kle	0.07 ± 0.04

Abbreviations: Bac, *Bacteroides*; Bif, *Bifidobacterium*; Bla, *Blautia*; Col, *Collinsella*; Ent, *Enterobacter*; Ery, *Erysipelotrichaceae UCG‐003*; Esc, *Escherichia‐Shigella*; Kle, *Klebsiella*; Lim, *Limosilactobacillus*.

**FIGURE 5:**
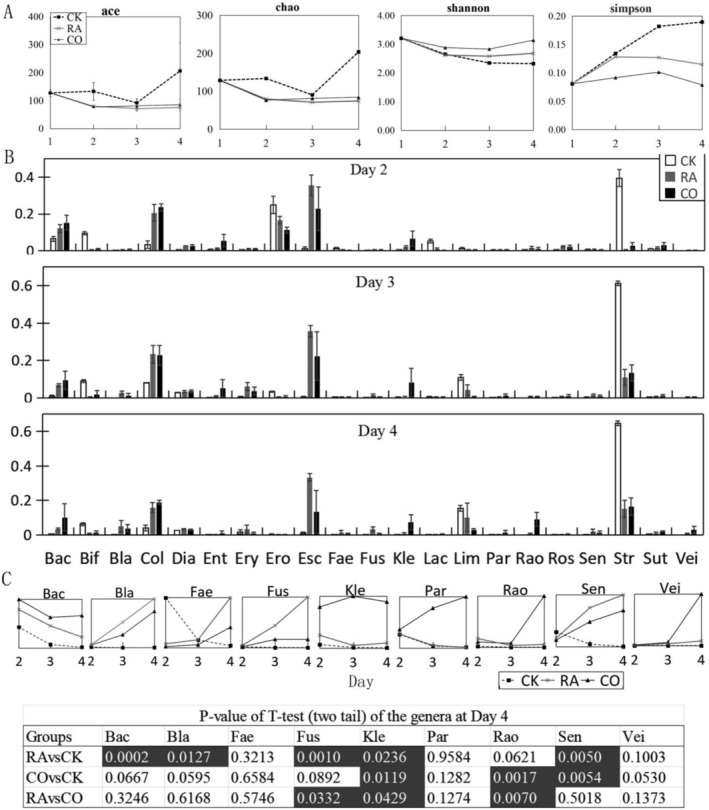
Influence of pecan kernels on gut microbiota. CO: Cooked nut; RA: Raw nut; CK: Without nut. (A) The bacterial diversity among groups. (B) Top 21 genera ranked by abundance variance during the Day 2–Day 4. (C) The comparison of abundance variation of nine most obvious genera from different treatment. Data were normalized based on the max abundance. Bac: *Bacteroides*; Bla: *Blautia*; Fae: *Faecalibacterium*; Fus: *Fusicatenibacter*; Kle: *Klebsiella*; Par: *Parabacteroides*; Rao: *Raoultella*; Sen: *Senegalimassilia*; Vei: *Veillonella*. White text in black background indicated significant differences in the genus between the two groups.

Microbiota diversity analysis (Figure 5A) indicated that while CK showed higher ACE and Chao1 indices (reflecting genus richness) by Day 4, RA and CO groups demonstrated superior Shannon indices, indicating greater evenness. This aligns with their higher number of dominant genera compared to CK, with CO most effectively enhancing the Shannon index. The CK's elevated Simpson index further confirmed reduced diversity due to disproportionate *Streptococcus* dominance. Although artificial medium limitations caused declining diversity in CK over time, the CO group uniquely induced higher diversity than initial samples by Day 4, underscoring cooked pecans’ role in promoting gut microbiota diversity.

Analysis of temporal changes in bacterial abundance identified 21 influential genera across treatment groups (Figure 5B), encompassing all 12 dominant bacteria from Table 2. This confirms that pecan supplementation substantially reshaped core microbiota. Notably, nine nondominant genera exhibited significant temporal and group‐dependent trends despite low baseline abundance, suggesting secondary ecological impacts.

Further examination of dynamically responsive genera (Figure 5C) revealed that raw pecans (RA) significantly increased *Blautia*, *Fusicatenibacter*, and *Senegalimassilia* from Days 2‐4. *Blautia*, which shows suppression of inflammation and adenoma reduction in colorectal cancer in animal models (Ni et al. 2022), showed progressive enrichment, rising 17‐fold within RA from Day 2 to Day 4 and reaching 193.8 times the control level (5.1%, *p* = 0.0127) to become dominant. *Fusicatenibacter*, which probably indirectly influences intelligence by regulating brain volume and has potential as a target for the treatment and prevention of cognitive impairments (Yao et al. 2024), surged to 1274.3 times control (3.3%, *p* = 0.001) and was uniquely elevated versus CO (5.6 times higher, *p* = 0.0332) by Day 4. *Senegalimassilia* (a probiotic with pancreatic cancer‐inhibiting properties (Jiang et al. 2023)) in RA attained 764.7 times control (2.0%, *p* = 0.005) by Day 4. Cooked pecans (CO) also induced significant *Senegalimassilia* enrichment but failed to promote *Fusicatenibacter* or *Blautia*. Instead, CO significantly promoted the growth of opportunistic pathogens, with *Klebsiella* abundance being notably higher in CO than in RA (*p* < 0.05) though increased in both groups relative to control. The opportunistic pathogen *Raoultella* (Appel et al. 2021) showed significant elevation exclusively in CO relative to both the control and RA (*p* < 0.05). Notably, *Bacteroides*, which facilitates bile acid metabolism for ulcerative colitis management (He et al. 2024), decreased in all groups but showed significantly attenuated reduction in RA compared to control (*p* = 0.0002). Although CO exhibited the slowest decline among groups, its difference versus control was not statistically significant (*p* = 0.067) due to high within‐group variability. In summary, the RA group significantly increased the proportions of *Bacteroides*, *Blautia*, *Fusicatenibacter*, *Klebsiella*, and *Senegalimassilia* in the gut microbiota, whereas the CO group significantly elevated *Klebsiella*, *Raoultella*, and *Senegalimassilia*. Notably, *Fusicatenibacter* was exclusively promoted by RA, while *Raoultella* was uniquely enhanced in CO. *Klebsiella* and *Senegalimassilia* increased significantly in both groups. Critically, RA demonstrated stronger promotion of bacteria associated with human health benefits compared to CO, which elevated opportunistic pathogens. Although this study highlights the significance of gut microbiota profiling for evaluating food nutritional value, future gut microbiota investigations across varying health backgrounds will help improve the methodological stability and better harness the application potential of this approach.

### 3.4 Metabolite Enrichment for Inflammation Inhibition and Neural Regulation in the Raw Group

**FIGURE 6:**
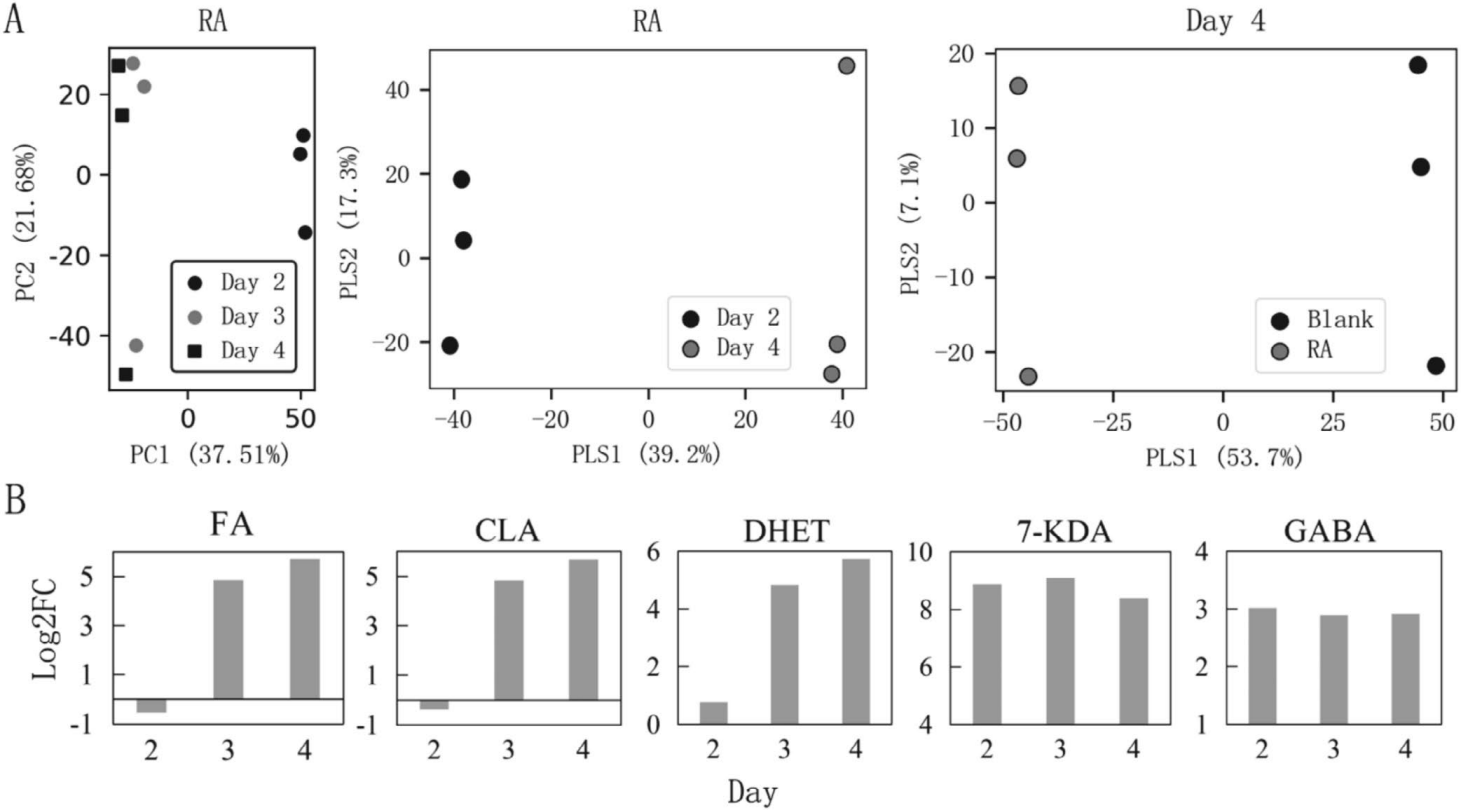
Integrative analysis of untargeted metabolomics data and differential metabolites in the RA group. (A) PCA (principal component analysis) of metabolomics data from samples of different groups, including PCA of metabolomics data from the RA group at different sampling times (from Day 2 to Day 4) (left), and PLS‐DA of metabolomics data between the Day 2 and Day 4 samples of RA treatment (middle) and PLS‐DA between RA and blank groups at Day 4 (right); (B) The changes over time of some annotated substances with the significant differences between the RA and blank. The Log2FC of *y*‐axis is log_2_ (RA/Blank). FA: (5*E*,9*E*)‐Farnesylacetone; CLA: 10*E*,12*Z*‐octadecadienoic acid; DHET: (+|‐)5,6‐DHET; 7‐KDA: 7‐Ketodeoxycholic acid; GABA: 4‐aminobutyric acid.

PCA analysis of untargeted metabolomics in samples from the RA group (raw pecan kernels) at different gut microbiota sampling time points indicated that the metabolomic profiles of Day 3 and Day 4 were relatively similar, both showing significant differences from Day 2 (PC1 = 64.46%). This not only demonstrates that the metabolomic characteristics of the RA group's microbiota were reshaped through transfer but also suggests that the metabolomic profiles of Day 4 samples gradually stabilized. Therefore, the metabolome of RA at Day 4 is suitable for prioritized research (Figure 6A). When comparing the metabolomes of the RA group between Day 4 and Day 2, eighty‐seven differential metabolites were identified from annotated metabolites, including 30 upregulated and 57 downregulated metabolites. In the comparison between the RA group and the Blank control group at Day 4, a total of 767 differential metabolites were identified, with 374 upregulated and 393 downregulated.

These differential metabolites are of significant importance for understanding the role of RA in this gut microbial system. For example (Figure 6B), the most significantly upregulated metabolite in the RA group from Day 2 to Day 4 was FA [(5E,9E)‐farnesyl acetone] (Log_2_(Day4/Day2) = 6.3, *p* = 0.0013). FA, a class of sesquiterpenoid substances, exhibits the ability to regulate cell membrane fluidity and lipid metabolism (Rodes et al. 1995). Next, CLA (10E,12Z‐octadecadienoic acid) also showed a marked increase postcultivation (Log_2_(Day4/Day2) = 5.9, *p* = 0.0010). As an isomer of linoleic acid, CLA possesses physiological activities such as antiobesity, antiatherosclerosis, and improved glucose tolerance, and can be produced by certain gut bacteria (e.g., *Enterococcus, Lactobacillus, Bifidobacterium*) via linoleate isomerase (LAI) (Iorizzo et al. 2024). DHET [(±)‐5,6‐dihydroxyeicosatrienoic acid] was another metabolite with rapid growth (Log_2_(Day4/Day2) = 4.9, *p* = 0.0019). DHET is generated from arachidonic acid under the action of cytochrome P450 enzymes and plays roles in regulating vascular tone, blood pressure, and inflammatory responses in vivo (Lu et al. 2001). In contrast to the above three metabolites, 7‐KDA (7‐ketodeoxycholic acid) and 4‐aminobutyric acid (GABA) did not show significant changes from Day 2 to Day 4, but their differences from the blank control were highly significant. The high content of 7‐ketodeoxycholic acid (a bile acid metabolite) in the culture system corresponded to high *Bacteroides/Blautia* abundance (He et al. 2024). Additionally, 4‐aminobutyric acid (GABA), a special nonprotein amino acid with crucial regulatory roles in the nervous system (Braga et al. 2024), was also significantly elevated in the RA group compared to the Blank control, aligning with the previously observed specific enhancement of intelligence‐related *Fusicatenibacter* in the RA group.

**FIGURE 7:**
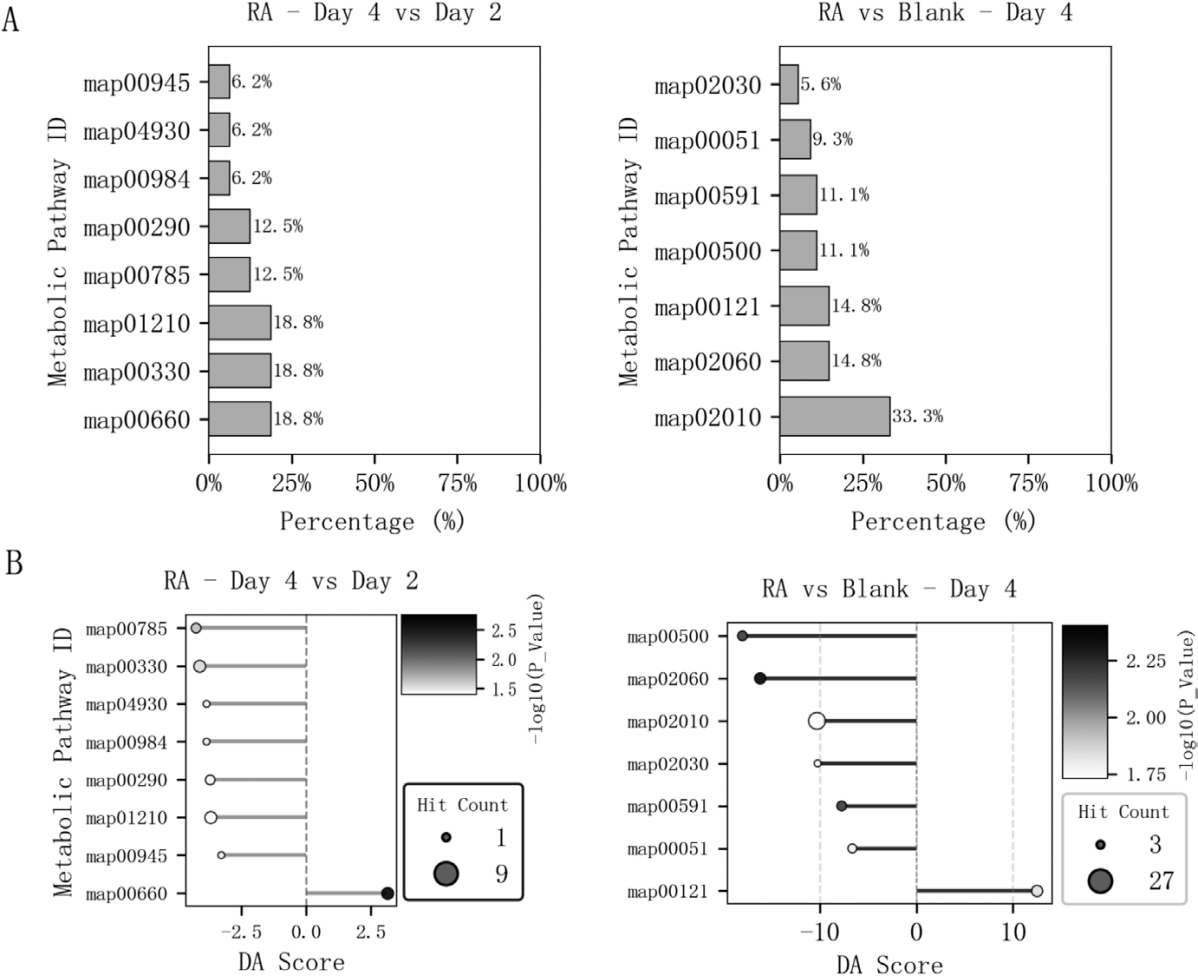
Enrichment analysis of metabolic pathways for differential metabolites in the RA group. (A) The classification diagrams of KEGG metabolic pathways of differential metabolites from different groups, including the diagram between the RA groups at Day 4 and Day 2 (left), and that between the RA and control (Blank) groups at Day 4 (right). The *x*‐axes represent the percentage of differential metabolites annotated under a specific pathway relative to the total annotated differential metabolites; (B) the DA score (differential abundance score) diagrams of KEGG metabolic pathways from different groups, including the diagram between the RA groups at Day 4 and Day 2 (left), and that between the RA and control (Blank) groups at Day 4 (right). Bubbles on the right of the central axis indicate upregulated pathway expression, while those on the left indicate downregulated. The metabolic pathways are as follows: map00051: Fructose and mannose metabolism, map00121: Secondary bile acid biosynthesis, map00290: Valine, leucine, and isoleucine biosynthesis, map00330: Arginine and proline metabolism, map00500: Starch and sucrose metabolism, map00591: Linoleic acid metabolism, map00660: C5‐branched dibasic acid metabolism, map00785: Lipoic acid metabolism, map00945: Stilbenoid, diarylheptanoid, and gingerol biosynthesis, map00984: Steroid degradation, map01210: 2‐oxocarboxylic acid metabolism, map02010: ABC transporters, map02030: Bacterial chemotaxis, map02060: phosphotransferase system (PTS), map04930: Type II diabetes mellitus.

Enrichment analysis of KEGG metabolic pathways for the abovementioned differential metabolites indicated (Figure 7A) that, when comparing Day 4 to Day 2, changes in differential metabolic pathways were primarily concentrated in C5‐branched dibasic acid metabolism (map00660), arginine and proline metabolism (map00330), and 2‐oxocarboxylic acid metabolism (map01210), followed by lipoic acid metabolism (map00785) and valine, leucine, and isoleucine biosynthesis (map00290). At the Day 4 stage, when comparing RA treatment to the Blank control, changes in metabolic pathways of differential metabolites were primarily concentrated in ABC transporters (map02010), followed by the phosphotransferase system (PTS) (map02060) and secondary bile acid biosynthesis (map00121). Further analysis of the DA Score for these metabolic pathways (Figure 7B) revealed that, when comparing Day 4 to Day 2, only C5‐branched dibasic acid metabolism (map00660) showed significant upregulation under RA treatment. A prior mouse study showed Cyclocarya paliurus polysaccharide (CP) primarily enriched C5‐branched dibasic acid metabolism (map00660) among KEGG pathways; similarly, our in vitro gut microbiota culture, using distinct materials and microbes, observed this pathway as the sole upregulated metabolic pathway (Wu et al. 2021). Additionally, at Day 4, RA treatment exhibited upregulation only in secondary bile acid biosynthesis (map00121) compared to the Blank control. This finding corresponds to the high content of metabolite 7‐KDA in the culture system, indicating that RA exerts a pronounced effect on bile acid metabolism. Collectively, RA exhibited metabolites and pathways supporting intestinal inflammation inhibition, immune regulation, and nervous system function, suggesting raw pecans may exert potential health benefits by modulating the gut microbiota‐metabolite axis, though their specific physiological effects require further validation in vivo (He et al. 2024).

## 4 Conclusions

This study developed a cost‐effective anaerobic culture method for evaluating food health impacts via standardized gut microbiota cultivation, integrating dynamic growth monitoring, ice‐induced dormancy transfers, and high‐throughput multiomics (16S rRNA sequencing + untargeted metabolomics) to enhance safety and reproducibility. Application showed *P. fermentans* significantly boosted Bifidobacterium abundance (4.6‐fold to 5.6‐fold vs. control) but reduced diversity. Raw pecan kernels (RA) promoted health‐associated genera (*Blautia*, *Fusicatenibacter*, *Bacteroides*) and metabolites (e.g., CLA, 7‐KDA), suggesting potential anti‐inflammatory and cognitive implications via microbiota modulation, with untargeted metabolomics revealing RA uniquely upregulated C5‐branched dibasic acid metabolism and secondary bile acid biosynthesis. Conversely, cooked kernels (CO) favored opportunistic pathogens (Klebsiella, Raoultella). These findings highlight gut microbiota profiling as critical for nutritional evaluation, though limitations include simplified cultures and homogeneous donors. Future studies with diverse samples and individual backgrounds will improve methodological stability and application potential.

## Author Contributions

**Xin‐Yue Liu:** data curation (equal), methodology (equal). **Ling‐Qin Zhu:** data curation (equal), methodology (equal), resources (equal). **Qun‐Ying Jin:** formal analysis (equal), investigation (equal), software (equal). **Hua‐Zheng Peng:** conceptualization (lead), methodology (equal), resources (equal), supervision (equal), writing – original draft (lead). **Tang‐Jun Zhu:** conceptualization (equal), funding acquisition (equal), supervision (equal), writing – review and editing (equal).

## Conflicts of Interest

The authors declare no conflicts of interest.

## Data Availability

The data that support the findings of this study are available from the corresponding author upon reasonable request.
